# Arabidopsis Plant Natriuretic Peptide Is a Novel Interactor of Rubisco Activase

**DOI:** 10.3390/life11010021

**Published:** 2020-12-31

**Authors:** Ilona Turek, Chris Gehring, Helen Irving

**Affiliations:** 1 Biomolecular Laboratory, Division of Biological and Environmental Sciences and Engineering, King Abdullah University of Science and Technology, Thuwal 23955-6900, Saudi Arabia; christophandreas.gehring@unipg.it or; 2 Department of Pharmacy and Biomedical Sciences, La Trobe Institute for Molecular Science, La Trobe University, Bendigo, VIC 3552, Australia; 3 Department of Chemistry, Biology and Biotechnology, University of Perugia, 06121 Perugia, Italy

**Keywords:** *Arabidopsis thaliana*, plant natriuretic peptide, plant peptidic hormone, phytohormone, rubisco activase

## Abstract

Plant natriuretic peptides (PNPs) are a group of systemically acting peptidic hormones affecting solute and solvent homeostasis and responses to biotrophic pathogens. Although an increasing body of evidence suggests PNPs modulate plant responses to biotic and abiotic stress, which could lead to their potential biotechnological application by conferring increased stress tolerance to plants, the exact mode of PNPs action is still elusive. In order to gain insight into PNP-dependent signalling, we set out to identify interactors of PNP present in the model plant *Arabidopsis thaliana*, termed AtPNP-A. Here, we report identification of rubisco activase (RCA), a central regulator of photosynthesis converting Rubisco catalytic sites from a closed to an open conformation, as an interactor of AtPNP-A through affinity isolation followed by mass spectrometric identification. Surface plasmon resonance (SPR) analyses reveals that the full-length recombinant AtPNP-A and the biologically active fragment of AtPNP-A bind specifically to RCA, whereas a biologically inactive scrambled peptide fails to bind. These results are considered in the light of known functions of PNPs, PNP-like proteins, and RCA in biotic and abiotic stress responses.

## 1. Introduction

Plants, due to their sessile lifestyle, have evolved sophisticated mechanisms to recognize and quickly respond to various signal inputs from the outside and to adapt to a constantly changing environment. As other multi-cellular organisms, plants also need to generate and respond to many internal signalling cues to organise their growth and development. Various biotic and abiotic stresses trigger highly complex stimulus-specific and systemic signals. The responses consist of different elements, including extra- and intracellular receptors, plant hormones, and secondary messengers. In particular, an increasing number of peptidic plant hormones, mainly discovered in the last 30 years or so, have been implicated in the modulation of diverse processes pertaining to both development and defence [1,2].

One group of such phytohormones are plant natriuretic peptides (PNPs), which are apoplastic and paracrine [3] stress response molecules [4,5] involved in maintaining salt and water homeostasis [5,6,7,8]. Expression of PNPs and their genes is elevated in response to osmotic stress [5], K^+^ starvation and pathogen attack [9], including hemibiotrophic *Pseudomonas syringae* pv. *tomato* (*Pst*) infection [10]. They have been localised in vasculature and isolated from xylem exudates [11], identified in apoplastic proteome [12], and shown to act as a systemic phloem signal, integrating responses to the whole plant [13]. In particular, an *Aradiposis thaliana* PNP, termed AtPNP-A (At2g18660; Q9ZV52), is implicated in many physiological processes ranging from the regulation of stomatal aperture [14], altering photosynthesis and leaf dark respiration rate [13], modulating osmoticum-dependent volume changes in protoplasts [6,14,15], regulation of developmental stage- and tissue-specific ion fluxes [7], and immune responses [10,16], including systemic acquired resistance [9,17].

Intriguingly, similar peptide signals are also made by phytopathogens to hijack specific functions of host plants. In the host–pathogens ‘arms race’, bacteria may acquire ‘eukaryotic-like’ genes from their hosts through horizontal gene transfer. A model example of such an ancient lateral gene transfer event [18,19] is found in the biotrophic pathogen *Xanthomonas axonopodis* pv. *citri*, a citrus canker agent which expresses a unique PNP-like gene, *XacPNP* [18]. The pathogen has been shown to modulate plant homeostasis to its own advantage by releasing XacPNP, a protein with an amino acid sequence similarity to PNPs and no homologues in other bacteria, and its synthesis is induced by nutrient-poor conditions in the apoplastic environments [20]. XacPNP mimics host PNP, resulting in significant changes to the proteome of the host, suppressing its immune response, and improving physiological conditions of the infected tissue, thus creating conditions favourable to pathogen survival [20,21,22]. Arabidopsis plants overexpressing *XacPNP* or *AtPNP-A* are more resistant to saline and oxidative stress and to *Pst* infection than control plants, while *PNP*-deficient plants are more susceptible [10]. Moreover, treatment of plant leaves with either AtPNP-A or XacPNP increases stomatal conductance and stomatal opening, concurring with higher leaf transpiration rates, induces starch reduction in guard cell chloroplasts, regulates leaf photosynthetic rates and efficiency of light utilization during photosynthetic CO_2_ fixation, and triggers increase in leaf dark respiration rates [13,20].

In analogy to vertebral natriuretic peptides, many effects exerted by PNPs involve rapid increases in 3′,5′-cyclic guanosine monophosphate (cGMP) [14,23]. A growing body of evidence indicates that reactive oxygen species (ROS) are also second messengers in the transduction of AtPNP-A signals [4,24]. Despite extensive efforts to elucidate molecular mechanism of AtPNP-A action over the last decade, only a handful of its interactors, including receptors [16,25], have been identified [26] and characterized [24].

In this study, we undertook a large-scale approach to identify novel binding partners of AtPNP-A using affinity-based isolation followed by liquid chromatography tandem mass spectrometric (LC-MS/MS) analysis. Our studies present evidence that rubisco activase (RCA; At2g39730; Q0WLM1) is a direct binding partner of AtPNP-A. RCA connects ATP hydrolysis with the structural remodelling of ribulose-1,5-bisphosphate carboxylase/oxygenase (Rubisco)—the principle enzyme of the Calvin–Benson–Bassham cycle of photosynthesis that fixes CO_2_ [27]. RCA facilitates the release of the inhibitory sugar phosphates from the active site of Rubisco, thereby sustaining Rubisco in its active state and continuing ribulose-1,5-bisphosphate (RuBP) substrate regeneration, which is a critical factor in photosynthesis, especially under heat stress conditions [28,29]. We also confirmed the specificity of the interaction between recombinant RCA and AtPNP-A proteins using surface plasmon resonance (SPR). These findings are discussed in the light of the currently known biotic and abiotic stress responses modulated by RCA, PNPs, and PNP-like proteins.

## 2. Materials and Methods

### 2.1. Plant Materials and Growth Conditions

Seeds of wild type (WT) *Arabidopsis thaliana* (Col-0) were surface-sterilized and vernalized, sown in Jiffy peat pellets and grown at 23 °C in 16 h of light (100 μmol s^−1^ m^−2^) per day.

### 2.2. Synthetic Peptides

**Figure 1:**
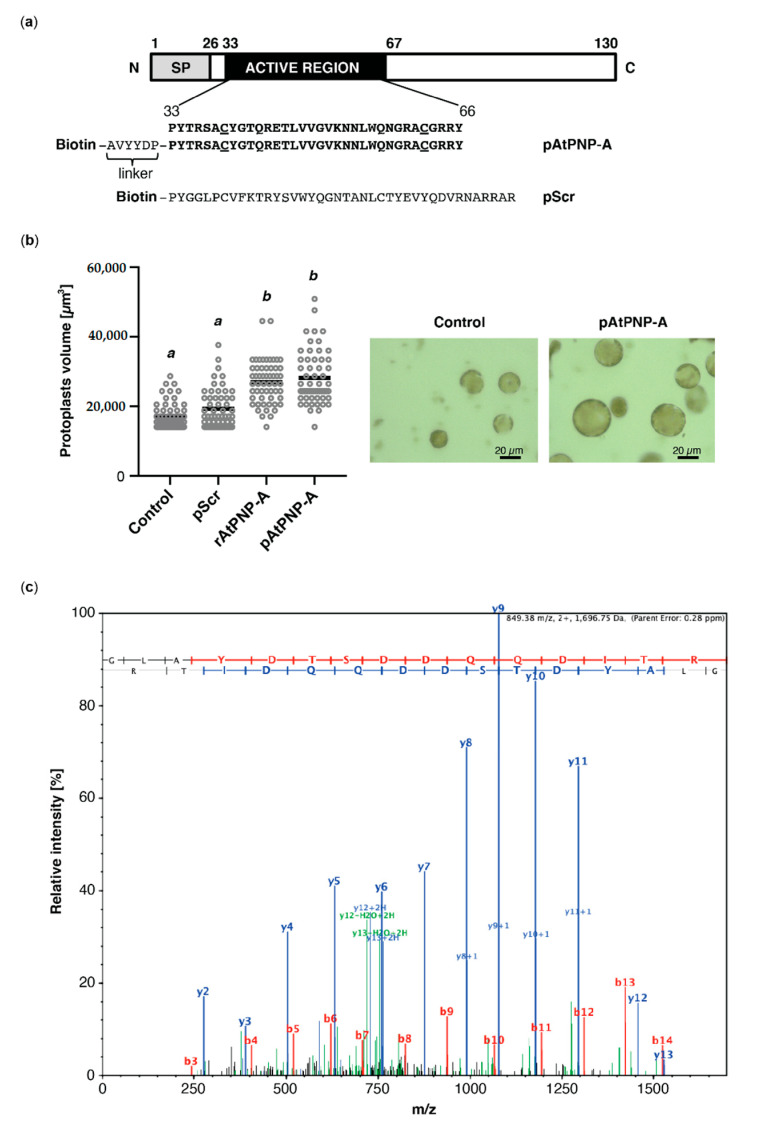
Biologically active peptide containing the active region of AtPNP-A binds to RCA. (**a**) Schematics showing domain organization of AtPNP-A and the amino acid sequence of N-terminally biotinylated peptides used in the affinity-based experiments. pAtPNP-A—a peptide containing the active site of AtPNP-A; pScr—the corresponding scrambled peptide; SP—signal peptide. Cysteine residues forming a disulfide bond are underlined. (**b**) Assaying biological activity of the pAtPNP-A and purified recombinant AtPNP-A (rAtPNP-A). *A. thaliana* (Col-0) mesophyll cell protoplasts suspended in 0.4 M mannitol were treated with either water, or 100 nM pScr (negative control), or with 100 nM pAtPNP-A, or with 1 μg mL^−1^ of rAtPNP-A protein for 20 min at room temperature. In each treatment, 50 randomly selected protoplasts with diameter >20 μm were included in quantitative analysis (scale bar = 20 μm). Protoplast volume was measured, and the data obtained from an exemplar experiment are plotted. Different superscript (*a* and *b*) indicate statistically significant results (mean ± SD, one-way ANOVA followed by Tukey–Kramer multiple comparison test, *n* = 50, *p* < 0.0001). (**c**) Exemplar MS/MS spectrum of a unique tryptic peptide of RCA (At2g39730) protein. N-terminal b ions and C-terminal y ions resulting from amide bond cleavage are labelled.

Peptides containing amino acid sequence of the active region of AtPNP-A and corresponding scrambled peptide, containing the same amino acid composition in a randomized order (Figure 1a), with or without N-terminal biotin tag, were purchased from GenScript (Piscataway, NJ, USA). The purity level (at least 95%) of the synthetic peptides was verified with HPLC.

### 2.3. Verification of Biological Activity of Synthetic Peptides

Biological activity of the synthetic peptides was verified in mesophyll cell protoplast (MCP) volume assay. MCPs were isolated from four-week-old WT plants [30]. The protoplasts were pelleted by 2 min centrifugation at 200× *g*, resuspended in osmotic solution [2 mL; 0.4 M mannitol 3 mM MES (2-(*N*-morpholino)ethane-sulphonic acid), 7 mM CaCl_2_, pH 5.7] and rested on ice for an hour before treatments. Responses of the protoplasts to water, 100 mM N-terminally biotinylated scrambled peptide (pScr) or AtPNP-A(33–66) peptide (pAtPNP-A), or the purified recombinant His-tagged AtPNP-A (1 μg mL^−1^) were assessed 20 min post-treatment at 20 °C. Each treatment was repeated at least three times with biological triplicates. MCPs were visualized by confocal microscopy (LSM780-NLO; Zeiss, Jena, Germany) and 50 randomly selected MCPs (diameter > 20 μm) were subjected to analysis for each treatment. Data are expressed as mean ± SD, and statistical analyses of one-way ANOVA followed by Tukey-Kramer multiple comparison test were performed using Prism 8.0 (GraphPad Software, San Diego, CA, USA).

### 2.4. Identification of RCA as an Interactor of AtPNP-A by Affinity-Based Isolation and LC-MS/MS Analysis

Isolation of putative interactors of AtPNP-A was performed as described previously [31] with minor modifications. For each sample, 5 g of plant material was used, no cross-linker was applied, the supernatant was incubated either with the N-terminally biotinylated pAtPNP-A or pScr (negative control) for 15 min. The protein extract mixture was incubated with Dynabeads M-280 (Life Technologies, Singapore) for 30 min. Eluted proteins were separated by SDS-PAGE (10% gels run for 15 min at 100V) and bands visualized with Coomassie Brilliant Blue (Figure S1). Bands were excised for trypsin in-gel digestion of proteins. MS analyses were performed on linear trap quadrupole (LTQ) Orbitrap Velos mass spectrometer (Thermo Scientific, Waltham, MA, USA) under conditions previously described [26]. Scaffold version 4.11.1 (Proteome Software Inc.) was used to validate MS/MS based peptide and protein identification. Peptide identifications were accepted if they could be established at greater than 95% probability by the Peptide Prophet algorithm. Protein identifications were accepted if they could be established at greater than 99% probability and contained at least two identified peptides. Protein probabilities were assigned by the Protein Prophet algorithm. Proteins that contained similar peptides and could not be differentiated based of MS/MS analysis alone were grouped to satisfy the principles of parsimony. Relative quantification of total spectrum counts of proteins identified with a confidence level of at least 99% at FDR < 0.1 in each sample containing pAtPNP-A or pScr (negative control) from three independent experiments was performed using Scaffold. Total spectra of the proteins identified in each of the three biological samples containing pAtPNP-A were compared to total spectra of the proteins identified in control samples and the differences were considered significant (*p* < 0.05) if the fold change by category (with a reference category being control) was greater than 2, verified by *t*-test with Bonferroni multiple test correction. The quantification file as well as the raw data are available via ProteomeXchange with identifier PXD023216, and the MS information is integrated in Supplementary Tables S1–S6.

### 2.5. Prediction of Protein Associations and Protein-Protein Docking

The structures of AtPNP-A was predicted using the iterative threading assembly refinement (I-TASSER; http://zhanglab.ccmb.umich.edu/I-TASSER/) method [32]. The crystal structure of *A. thaliana* RCA (4W5W) [33] was derived from RCSB PDB, https://www.rcsb.org/). Protein–protein docking was completed with ClusPro (version 2.0; http://cluspro.bu.edu/publications.php) [34], and models were analysed and visualized with UCSF Chimera (version 1.10.2) [35].

### 2.6. Expression and Purification of Recombinant Proteins

Expression of the N-terminally 6xHis-tagged AtPNP-A and RCA proteins was conducted in BL21 (DE3) One Shot *E. coli* cells (Life Technologies). The recombinants were purified by affinity chromatography with Ni-NTA beads (Qiagen, Venlo, the Netherlands) and HisTrap HP column (GE Healthcare, Chicago, IL, USA) as previously described [25]. The purity of protein preparations was verified on 12.5% SDS-PAGE stained with Coomassie Brilliant Blue (Bio-Rad, Hercules, CA, USA). Identity of the recombinants was confirmed in MS analysis, and protein concentration was determined by the Bradford method using bovine serum albumin (BSA) as a standard.

### 2.7. Surface Plasmon Resonance (SPR) Analyses

SPR experiments were performed at 20 °C on a Biacore T100 instrument (GE Healthcare LifeSciences) with the use of Series S CM5 on NTA sensor chips as previously described [25]. Data were acquired with Biacore T100 control software (version 2.0.2, GE Healthcare LifeSciences, Chicago, IL, USA). Kinetic analyses were performed at the flow of 100 μL min^−1^ with pAtPNP-A (at 3.78 μM and consecutive two-fold dilutions; 11 injections included) employed as a ligand, whereas recombinant His-tagged RCA was used as an analyte immobilized on the active surface of the series S CM5 sensor chip with amine coupling kit. The surface was regenerated with glycine solution, pH 2.0 (GE Healthcare LifeSciences). The final sensorgram was generated with Scrubber (BioLogic Software Pty Ltd., Campbell, Australia).

## 3. Results

### 3.1. Identification of RCA as an Interactor of AtPNP-A

In order to identify interactors of AtPNP-A, we performed affinity-based isolation of proteins binding to biologically active AtPNP-A(33–66) bait. Since preceding investigations have delineated the active domain of AtPNP-A [14], we synthesized an N-terminally biotinylated peptide (pAtPNP-A) containing this active region of the protein, which encompasses the conserved disulfide bond, and a scrambled peptide (pScr), with the same amino acid composition as pAtPNP-A but assembled in random order (Figure 1a). Biological activity of both peptides, as well as the purified N-terminally His-tagged AtPNP-A protein (rAtPNP-A), was verified in mesophyll cell protoplast (MCP) volume assay. The effect exerted by pAtPNP-A, but not pScr, on MCPs was comparable to the protoplast volume increase caused by treatment with equimolar amounts of purified rAtPNP-A (Figure 1b). Since responses of MCPs to the pScr were negligible, the biological activity of both pAtPNP-A and rAtPNP-A was ascertained.

Both peptides were subsequently used as baits in the affinity isolation of putative interactors of AtPNP-A. Protein extracts from *A. thaliana* (Col-0) plants were incubated with either pAtPNP-A or pScr peptide and potential interactors of AtPNP-A were isolated through biotin–streptavidin interaction. Experiments with the use of pScr peptide were aimed at identification and exclusion of nonspecific interactors. The eluted proteins were separated by SDS-PAGE, and then excised bands underwent in-gel protein digestion with trypsin before LC-MS/MS identification of the tryptic peptides and subsequent relative quantification of spectral counts corresponding to peptides matching putative binding partners of pAtPNP-A (Table 1). Among the proteins found to physically interact with AtPNP-A was rubisco activase (RCA) which was identified with high confidence (Table 1 and Figure 1c).

### 3.2. RCA Binds to Recombinant AtPNP-A In Vitro

**Figure 2:**
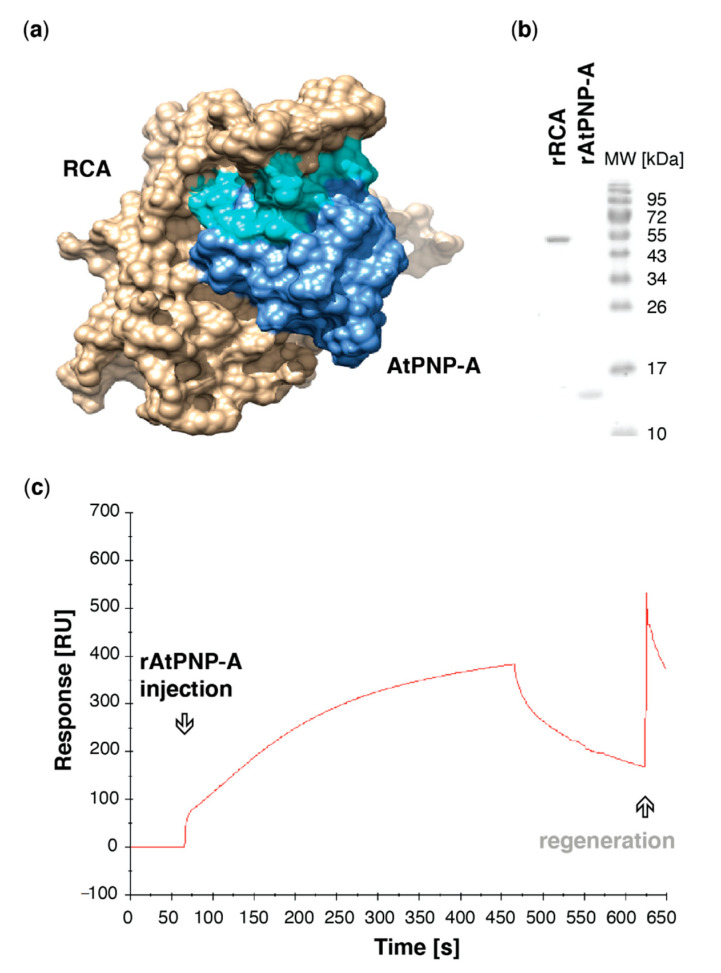
AtPNP-A directly interacts with Arabidopsis RCA in vitro. (**a**) Molecular docking of AtPNP-A and RCA monomer. Surface model depicts predicted docking of AtPNP-A (blue), with its active region (cyan), and RCA (tan). The structure of AtPNP-A was predicted using the iterative threading assembly refinement (I-TASSER; version 5.1) method [32], while the 4W5W crystal structure of *A. thaliana* RCA [33] was derived from RCSB PDB. Protein—protein docking was performed using ClusPro) [34]. The models were analyzed and visualized using UCSF Chimera (version 1.10.2) [35]. (**b**) Recombinant protein preparations used in SPR analyses. (**c**) Exemplar sensorgrams depicting referenced binding response of the recombinant RCA and AtPNP-A proteins. Purified rRCA was immobilized using amine-coupling chemistry on the active surface of the CM5 sensor chip, while the reference surface was blank activated and did not carry any ligand, and the purified rAtPNP-A was used as an analyte.

To gain insight into potential binding sites, protein docking simulations using the crystal structure of A. thaliana RCA monomer [33] and predicted 3D structure of AtPNP-A pointed at an interaction between these proteins occurring at active site of AtPNP-A (Figure 2a). AtPNP-A was predicted to bind in the grove formed by α-helix 2 (H2), which faces the C-terminal domain of the AAA^+^ module [33], and α-helices 8 and 9. To assess whether AtPNP-A binds to RCA, the proteins were expressed as N-terminally His-tagged recombinants, purified (Figure 2b), and in vitro binding of the recombinant proteins was verified using surface plasmon resonance (SPR), where rAtPNP-A bound to the rRCA protein immobilized on the active surface (Figure 2c).

### 3.3. Active Site of AtPNP-A Determines Specificity of Binding to RCA

**Figure 3:**
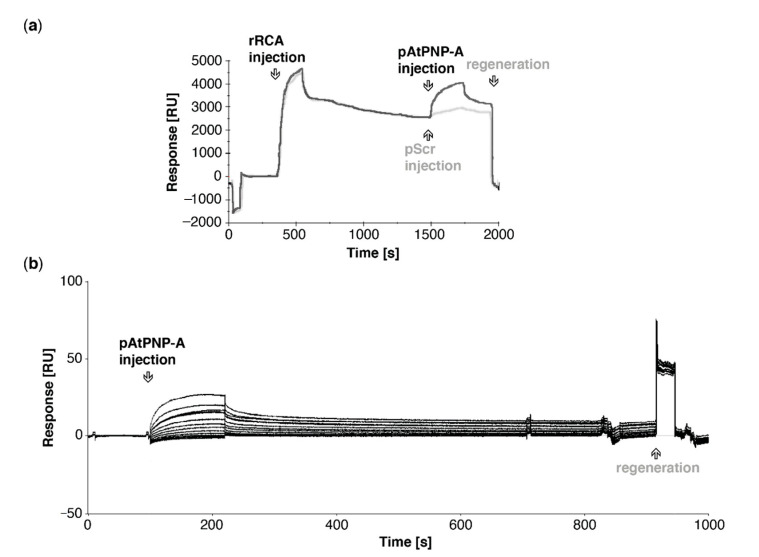
In vitro association of AtPNP-A active region (pAtPNP-A) with recombinant RCA (rRCA). (**a**) Exemplar sensorgrams depicting referenced binding response of pAtPNP-A (black line) or pScr (grey line) with rRCA immobilized on the active surface of the NTA sensor chip. Reference surface of the NTA chip was not modified, according to the manufacturer’s instructions, and did not carry the recombinant protein. In both analyses the ligand was immobilized at the same levels, analytes were injected at the same concentration and conditions of runs kept constant. (**b**) Exemplar sensorgrams depicting referenced binding response in kinetic analysis of binding between pAtPNP-A (3.78 μM and consecutive two-fold dilutions, as in Table 3) and rRCA immobilized on the active surface of the CM5 sensor chip. Reference surface of the NTA chip was not modified, according to the manufacturer’s instructions, and did not carry any protein.

*In silico* docking predicted the active region of AtPNP-A being a predominant site of RCA binding (Figure 2a), which is consistent with our affinity-based isolation of RCA as an interactor of pAtPNP-A. To assess whether the biologically active region of AtPNP-A is sufficent to bind RCA, we probed binding of the pAtPNP-A to the immobilized rRCA. As shown in Figure 3a, the active region of AtPNP-A is sufficient for binding of recombinant RCA. In order to eliminate the possibility of a nonspecific interaction between pAtPNP-A and RCA resulting from a charge effect residing in the primary structure of the peptide, specificity of the binding between the bio-active region of AtPNP-A and purified rRCA was examined. The analyses made use of the NTA sensor chip with immobilized His-tagged rRCA and the biologically active pAtPNP-A or the corresponding biologically inactive pScr peptide were injected at the same concentration through the flow cells. This results in significant binding of the pAtPNP-A and only negligible accumulation of pScr (Figure 3a), confirming the specificity of the interaction between pAtPNP-A and rRCA. Kinetic analysis (Table 2) of the binding between pAtPNP-A and rRCA reveals strong association, with the dissociation constant (K_D_) in the sub-micromolar range (5.3 × 10^−8^ M) (Figure 3b, Table 3).

## 4. Discussion

Understanding of the signalling pathways that underlie plant stress responses is a key goal in the effort toward developing plants with increased resistance to adverse environmental conditions. Abiotic stresses, including heat, drought, and salinity, imposed on plants are more potent than in the past and affect plant photosynthetic processes and development, translating into poor yield. Rubisco, the CO_2_-fixing enzyme of the reductive pentose phosphate pathway, can be one of the major limitations to the rate of leaf photosynthesis. Rubisco and its activation by RCA are important potential targets for crop improvement, particularly with respect to photosynthesis during changes in light environment [36]. Although 90–95% of crop yield is produced by photosynthesis, increasing crop production in order to meet the rising demands for food by the growing world population will largely rely on improving photosynthesis [37,38].

Here, we described the identification of RCA, which stimulates the activation state of Rubisco, as a novel direct binding partner of AtPNP-A (Figure 1c). Confidence in our affinity chromatography isolation and subsequent mass spectrometric identification of RCA as a protein binding to the biologically active site of AtPNP-A (Figure 1a) has been strengthened by the fact that the total spectrum counts for RCA peptides in samples where the biologically active pAtPNP-A was used as a bait is significantly greater than in the negative control samples where the pScr scrambled peptide (Figure 1a) was used (Table 1). This indicates that the binding is driven by the three-dimensional structure of the peptide, as predicted in our molecular docking analyses (Figure 2a), rather than the non-specific binding effects caused purely by the amino acid composition of the peptide. Binding between the purified recombinant AtPNP-A and RCA (Figure 2b) was confirmed by us in vitro (Figure 2c). SPR kinetic analyses revealed strong and concentration-dependent association of pAtPNP-A with rRCA (Table 2), with dissociation constant in nanomolar range (Figure 3b, Table 3), whereas the binding of the pScr was negligible (Figure 3a).

It is important to note that RCA has been previously identified as a strong interactor of the AtPNP-A protein (lacking the signal peptide) in the Yeast Two-Hybrid assay [24], which is in accordance with our kinetic analyses. Furthermore, RCA was also proposed as a putative binding partner of pAtPNP-A based on cross-linking experiments performed in MCPs, followed by affinity chromatography and mass spectrometric identification [24,26]. Finally, our recent study indicates AtPNP-A localizes into chloroplasts [24], which may further support potential biological relevance of its interaction with a stromal RCA protein. Although the data reported here are of preliminary nature and the biological significance of this interaction is still to be elucidated, this increasing body of evidence has potential implications of the interaction between PNPs and RCA for plant development and stress responses. RCA has been identified as an oxidation target of thioredoxin-like2 and associated with oxidative thiol modulation in chloroplasts through the ferredoxin-thioredoxin reductase/thioredoxin [39]. It is possible that PNPs, proteins that contain a redox-active Cys pair (Figure 1a) are also—directly or indirectly—involved in the redox regulation of proteins in chloroplasts, in order to adjust chloroplast physiology as changes in light environments occur, in particular while resting their photosynthetic activity at night.

RCA gene and/or protein expression is affected by biotic [40] and abiotic [41] conditions in various species. In Arabidopsis, which expresses two RCA isoform products of alternative splicing [42], moderate reductions of *RCA* decrease growth and above ground biomass and leaf area [43], while the absence of high levels of soluble sugars due to cellulose biosynthesis inhibition can reduce *RCA* expression in Arabidopsis seedlings [44]. In tobacco (*Nicotiana attenuata*), silencing the expression of *NaRCA* decreased photosynthetic capacity and biomass [45]. Similarly, PNPs, including AtPNP-A, are well documented to modulate plant responses to biotic [17,20] and abiotic stresses [9,14]. Thus, it is possible some of these plant responses are due to the novel interaction between RCA and PNPs that we describe here.

RCA transcript and protein level is down-regulated by jasmonic acid (JA) in a coronatine-insensitive 1 (COI1)-dependent manner and correlated with JA- and dark-induced leaf senescence [46]. Interestingly, AtPNP-A is a senescence-enhanced gene [9] and premature senescence is displayed by *atpnp-a* knock-down mutant plants (data not shown). Tobacco (*Nicotiana tabacum*) *RCA* gene is involved in resistance to herbivory, and its down-regulation impaired JA signaling and led to reduced defense against both generalist (*Spodoptera littoralis*) and specialist (*Manduca sexta*) herbivore elicitation [45]. Moreover, caterpillar-specific post-translational modifications of RCA were detected in *A. thaliana* plants exposed to herbivory by fourth instar *Spodoptera exigua* [47]. Since plants gain resources via photosynthesis and allocate them to growth, reproduction, and defence, how resources are allocated to defence and other sinks is of particular importance for plant survival. Modulation of photosynthetic capacity of the plant by attacking pathogens is crucial for successful infection by microbes and insects alike. Interestingly, elevated RCA protein was observed 30 min post-treatment in protein extracted from leaves of citrus plants treated with XacPNP compared with mock-treated plants, which could indicate XacPNP-induced increase in anabolism, most likely resulting in net solute gain in the affected tissue [21]. Moreover, although host RCA protein level decreases upon infection with *X. axonopodis* pv. *citri*, compared with non-infected plants, infection of citrus leaves with the strain expressing XacPNP leads to a lower decrease in RCA level compared to the infection with the *XacPNP* deletion strain. This finding implies that XacPNP prevents reduction of host photosynthetic proteins, including RCA, efficiently counteracting the shut-down of plant photosynthesis [22]. Of note, exogenous application of AtPNP-A peptide enhanced susceptibility to *Pst* DC3000 [16]. AtPNP-A was shown to modulate leaf photosynthetic rates and apparent photon yield, thus regulating efficiency of light utilization during photosynthetic CO_2_ fixation [20]. It is conceivable that these changes in photosynthesis parameters due to PNPs action are mediated by its direct interaction with RCA, as revealed by us in this study. Due to the lack of biological studies, this speculative statement still requires experimental evidence; it would be interesting to elucidate the molecular mechanism of this process by phenotypical characterization and measurement of gas exchange in *atpnp-a*, *rca*, and double knock-down and overexpressing Arabidopsis mutant plants. For instance, overexpression of *RCA* gene in the *A. thaliana* silique wall enhanced the photosynthetic activity of the tissue and resulted in significantly increased seed weight and oil content [38]. In the light of our findings, it is tempting to speculate PNPs interaction with RCA may play a role in modulating photosynthetic capacity also in this tissue and may have implications for breeding efforts focused on improving seed and oil production.

RCA levels were proposed to play an important role in maize and wheat productivity under supra-optimal temperature conditions [48], while salt exposure significantly up-regulated phosphorylation of RCA [49]. Therefore, it would be of interest to verify whether AtPNP-A has any impact on the transcript and/or protein level of RCA (and vice versa) or its ability to undergo post-translational modifications. Of note, plants overexpressing *AtPNP-A* were more resistant to saline and oxidative stress than *AtPNP-A* deficient lines [10].

Finally, RCA is a thermolabile protein [50], and heat-deteriorated RCA is incapable of maintaining Rubisco in its active form, thus binding of PNPs to RCA may modulate thermostability of RCA. Such a mechanism for protecting RCA from thermal denaturation during heat stress and allowing acclimation of photosynthesis to heat stress, has been proposed for its interaction with chaperonin-60β [51]. It is also conceivable that AtPNP-A interaction with RCA may change its susceptibility for being post-translationally modified [52] (e.g., phosphorylation at Thr78 serves a negative regulatory role [53]) or its ability to oligomerize and proneness to proteolytic degradation. Detailed biochemical and physico-chemical characterization of the AtPNP-A interaction with RCA would provide insight into these aspects of the interaction.

## Figures and Tables

**Table 1:** Proteins identified as putative interactors of AtPNP-A in affinity-based isolation followed by liquid chromatography tandem mass spectrometric (LC-MS/MS) analyses.

Protein Name	Accession Number (TAIR)	*t*-Test (*p*-Value < 0.05)
Actin 7 (ACT7)	AT5G09810.1	<0.00010
Rieske (2Fe-2S) domain-containing protein	AT1G71500.1	0.00020
Photosystem I light harvesting complex gene 3 (LHCA3)	AT1G61520.1	0.00045
Photosystem I subunit G (PSAG)	AT1G55670.1	0.00056
Phosphoglycerate kinase 1 (PGK1)	AT3G12780.1	0.00061
RAB GTPase homolog E1B (RABE1b)	AT4G20360.1	0.00061
Glyceraldehyde-3-phosphate dehydrogenase B subunit (GAPB)	AT1G42970.1	0.00096
Protochlorophyllide oxidoreductase C (PORC)	AT1G03630.1	0.00098
Rubisco activase (RCA)	AT2G39730.2	0.0011
Light harvesting complex of photosystem II 5 (LHCB5)	AT4G10340.1	0.0019
GTP binding Elongation factor Tu family protein	AT1G07920.1	0.0024
Actin 8 (ACT8)	AT1G49240.1	0.0027
Eukaryotic translation initiation factor 4A1 (EIF4A1)	AT3G13920.1	0.0045
Cobalamin-independent synthase family protein (ATMS1)	AT5G17920.1	0.0055
Photosystem II reaction center protein C (PSBC)	ATCG00280.1	0.0058
CLPC homologue 1 (CLPC1)	AT5G50920.1	0.0063
ATP synthase subunit alpha (ATPA)	ATCG00120.1	0.0092
Carbonic anhydrase 2 (CA2)	AT5G14740.1	0.012
Photosystem I light harvesting complex gene 1 (LHCA1)	AT3G54890.1	0.013
Photosynthetic electron transfer C (PGR1)	AT4G03280.1	0.014
Chlorophyll A/B binding protein 3 (CAB3)	AT1G29910.1	0.014
ADP/ATP carrier 1 (AAC1)	AT3G08580.1	0.016
Protochlorophyllide oxidoreductase B (PORB)	AT4G27440.1	0.018
FtsH extracellular protease family (FTSH5)	AT5G42270.1	0.018
Plastid transcriptionally active 16 (PTAC16)	AT3G46780.1	0.019
Glutathione S-transferase phi 8 (GST6)	AT2G47730.1	0.019
Tubulin/FtsZ family protein (TUA6)	AT4G14960.2	0.019
Mitochondrial substrate carrier family protein	AT5G19760.1	0.026
Photosystem I subunit F (PSAF)	AT1G31330.1	0.027
Chaperonin 60 beta (LEN1)	AT1G55490.1	0.035
Transketolase	AT3G60750.1	0.038
Glyceraldehyde 3-phosphate dehydrogenase A subunit (GAPA)	AT3G26650.1	0.038

Relative quantification of total spectrum counts of proteins identified with a confidence level of at least 99% at FDR < 0.1 in each sample containing pAtPNP-A or pScr (negative control) from three independent experiments using Scaffold Q+ program. The MS data are available via ProteomeXchange with identifier PXD023216 and integrated in Tables S1–S6.

**Table 2:** Parameters of the binding cycles.

Concentration [M]	RI [RU]	SE (RI)
7.390 × 10^−9^	0.7	0.026
1.478 × 10^−8^	1.4	0.026
2.956 × 10^−8^	2.4	0.026
5.912 × 10^−8^	3.5	0.026
1.182 × 10^−7^	4.8	0.026
2.365 × 10^−7^	5.5	0.027
4.730 × 10^−7^	6.4	0.027
9.459 × 10^−7^	7.6	0.028
1.892 × 10^−6^	9.7	0.028
3.784 × 10^−6^	14.3	0.029
7.390 × 10^−9^	0.9	0.026

RI—refractive index, SE—standard error, RU—resonance unit.

**Table 3:** Kinetic parameters of the interaction between pAtPNP-A and rRCA.

Kinetics Parameter	Value
k_a_ [1/Ms] (±SE)	1.630 × 10^4^ (±41)
k_d_ [1/s] (±SE)	8.517 × 10^−4^ (±3.7 × 10^−6^)
K_D_ [M]	5.226 × 10^−8^
R_max_ [RU] (±SE)	13.2 (±0.17)
Flow [μL/min]	100
Chi^2^ [RU^2^]	0.815
U-value	5
tc	1.903 × 10^14^
k_t_ [RU/Ms]	5.427 × 10^7^

k_a_—association rate constant, k_d_—dissociation rate constant, K_D_—equilibrium dissociation constant, R_max_—maximum response units reached at equilibrium, k_t_—mass transfer constant, tc—flow-rate independent component of the mass transfer constant, Chi^2^—average squared residual, U-value—uniqueness value for kinetic rate constants, SE—standard error, RU—resonance unit.

## Supplementary Materials

The following are available online at https://www.mdpi.com/2075-1729/11/1/21/s1. Complete proteomics dataset was deposited to the ProteomeXchange Consortium via the PRIDE partner repository with the dataset identifier PXD023216 and 10.6019/PXD023216.; Figure S1. Image of elution fractions from the affinity chromatography experiment separated on 10% SDS-PAGE run for 15 min at 100V and stained with Coomassie Brilliant Blue.; Table S1. Peptide quantitative report.; Table S2. Peptide report.; Table S3. Protein report.; Table S4. Samples report.; Table S5. Spectrum count report.; Table S6. Spectrum report.
