# Supplementary material for: Arabidopsis Plant Natriuretic Peptide Is a Novel Interactor of Rubisco Activase
Source: Life (Basel). 2020 Dec 31;11(1):21. doi: 10.3390/life11010021 (PMC7823470; doi:10.3390/life11010021)
Supplement: Supplementary file 1 [file life-11-00021-s001.zip › life-1038772-supplementary/Supplementary material/Supplementary Materials-FigS1.docx]

*Supplementary Materials*

Arabidopsis Plant Natriuretic Peptide Is a Novel Interactor of Rubisco Activase

Ilona Turek ^1,2,^*, Chris Gehring ^1,3^, and Helen Irving ^2,^*


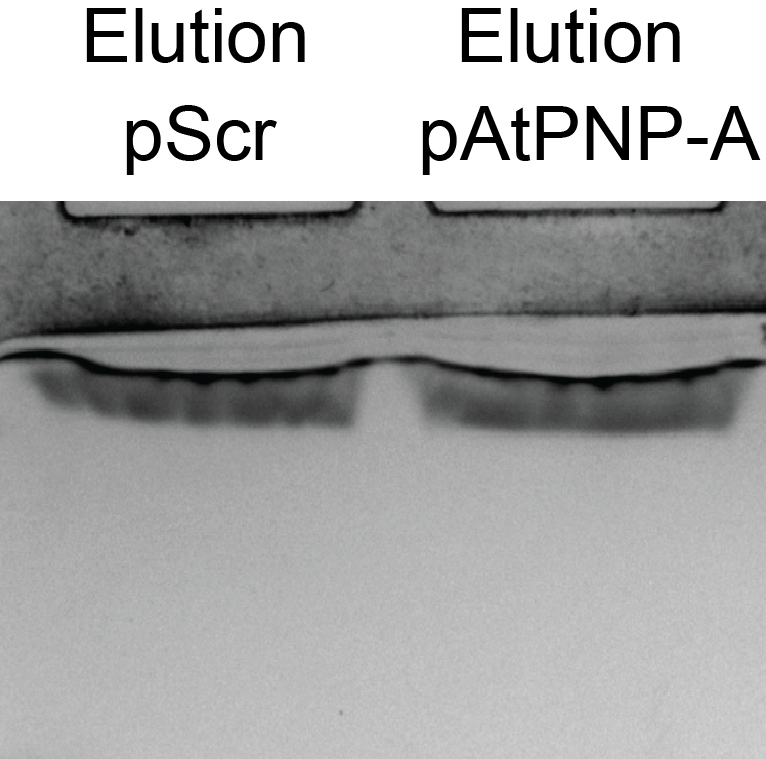


**Figure S1.** Image of elution fractions from the affinity chromatography experiment separated on 10% SDS-PAGE run for 15 min at 100V and stained with Coomassie Brilliant Blue.
